# Heart Disease in Mothers of Children with Duchenne Muscular Dystrophy

**DOI:** 10.2174/011573403X292850240719074112

**Published:** 2024-07-23

**Authors:** Rose Mary Ferreira Lisboa da Silva

**Affiliations:** 1 Department of Internal Medicine - Faculty of Medicine, Federal University of Minas Gerais, Belo Horizonte, Minas Gerais, Brazil

**Keywords:** Heart disease, Duchenne muscular dystrophy, female carriers, cardiomyopathy, health management

## Abstract

Female carriers of Duchenne Muscular Dystrophy (DMD) carry a heterozygous pathogenic variant in the dystrophin gene and can transmit pathogenic variants to their offspring. DMD is an X-linked recessive disease that affects up to 19.8 in every 100,000 male births. Those carriers with symptoms can be referred to as women with dystrophinopathy. Even among asymptomatic carriers, cardiac involvement can be verified in between 2.5% and 75% through echocardiography. The most commonly affected wall of the left ventricle is the inferolateral, with myocardial fibrosis detected by cardiac nuclear resonance. Therefore, screening is recommended for these women carriers due to the risk of cardiomyopathy. There is a lack of longitudinal studies on the evolution of these carriers. In this article, data on clinical presentation, cardiac assessment for female patients with dystrophinopathy and DMD carriers, and approaches for these patients are discussed.

## 1 INTRODUCTION

Duchenne Muscular Dystrophy (DMD) is an X-linked recessive mutation, a progressive muscular degenerative disease with a prevalence of 8.29 per 100,000 men. It rarely occurs in women, less than 1 per million, usually with autosomal translocations, Turner syndrome [1, 2]. The prevalence at birth can reach 19.8 per 100,000 men due to non-survival beyond paediatric age [3]. Through genetic testing and/or muscle biopsy, the global prevalence was 16.2 per 100,000, with a sample size of 1654,670 people [4].

Although there have been contributions from Bell, Semmola, Conte, Gioja, Partridge, and the clinical and pathological description of DMD by Edward Meryon, the disease received the eponym associated with the French neurologist Guillaume-Benjamin-Amand Duchenne for his publications in 1868. It was up to British neurologist Sir William Richard Gowers to describe and illustrate the maneuver used by the patient with DMD to stand up, which was described as Gowers' sign [5-7].

As it is an X-linked recessive disease, women with 1 X chromosome that carries the mutation have a 50% chance of transmission with each pregnancy. Most mutations are hereditary. However, in 30% of cases, mutations are spontaneous [8, 9]. About two-thirds of mothers of male patients with DMD and no family history are heterozygous. A proband woman, which occurs rarely, may have inherited the pathogenic variant from her mother or father, or the pathogenic variant may be de novo [9]. In rare cases, the DMD variant is inherited by the proband's asymptomatic father with deletions of low penetrance and expression as an incidental finding of copy number variants located in X-linked genes in girls [10, 11]. Therefore, there is a need for prenatal screening, genetic counseling, and an integral approach for carrier mothers.

In a recent workshop [12], there was a recommendation to use the term “carrier of the pathogenic variant of the DMD gene” or “DMD carrier” for women without symptoms or signs and who carry a pathogenic variant of the DMD gene. For women with clinical findings, the recommended term was “patients with dystrophinopathy,” and the term “manifest carrier” should be avoided. Therefore, in this mini-review, data on clinical presentation, cardiac assessment for female patients with dystrophinopathy and DMD carriers, and approaches for these patients are discussed.

## 2 CLINICAL PRESENTATION

Recent data, with a cohort of 460,608 individuals from 11 ethnicities and evaluating a total of 176 genetic conditions, demonstrated that the prevalence of DMD carriers, including also Becker muscular dystrophy, was 1 in 813 women [13]. A nationwide genetic carrier screening program for DMD was also recently published, and a 1:1046 prevalence of clinically significant variants in the DMD gene was found in 85,737 tests [14]. This DMD carrier rate could reach 1:2500, considering the frequency of the disease in men is 1:5000. However, the prevalence of DMD carriers depends on the screening method. A study with 12,362 women demonstrated that the rate of carriers of large pathogenic deletions or duplications, all involving multiple exons, was 1:1374 [15]. Given that about 30% of DMD cases are de novo pathogenic variants, carrier screening and its triage technique are crucial.

The majority of these women will be asymptomatic, that is, DMD carriers, but these carriers may develop muscular symptoms or cardiac involvement, becoming patients with dystrophinopathy. The postulated mechanism for DMD carriers to become patients with dystrophinopathy is attributed to the extent of random X-inactivation of the normal X chromosome *versus* the dystrophic X chromosome [8, 16, 17].

The incidence of cardiac involvement in DMD carriers ranged from 8.5% to 12% across 7 articles extracted from a review of 1,002 reports published from 1967 to 2017 in MEDLINE [18]. The sample ranged from 56 to 152 women with the mutation and with a mean age between 32.8 and 45 years, with assessment using electrocardiogram and echocardiogram. However, there are studies that have demonstrated a proportion of up to 56.2% of myocardial damage in 152 women with the DMD gene [19] or up to 2/3 of 52 women through electrocardiogram and/or echocardiography [20]. In a study of 16 women with the DMD gene, among whom 31% were symptomatic, there were signs of left ventricular dysfunction in 75% of cases [21]. Symptoms of muscular impairment such as weakness, myalgia, cramps, and exercise intolerance occur in a higher proportion in 81% of these women [22].

There is a progression of myocardial damage with age, with the detection of some cardiac abnormality in up to 58.3% up to 15 years of age and up to 93.8% in those over 50 years of age [19]. Thus, the age of onset of symptoms or cardiac involvement occurs mainly from the fourth decade of life [18, 23, 24]. When comparing carrier DMD and patients with dystrophinopathy, the age was lower in those with symptoms, especially muscular symptoms, and with increased serum creatine kinase (CK) levels [24, 25].

Symptoms related to cardiac involvement are dyspnea, palpitations, precordial compression, and edema [26-28]. These symptoms may be present in 25.7% [25] to 36% [20-23, 26] of women carrying the DMD gene. As previously reported, there is an association between age and symptoms [18, 19, 23, 24]. However, there is no genotype-phenotype correlation [29].

Despite the asymptomatic course in most women with the DMD mutation and symptoms in up to about a third of those with dystrophinopathy, cases of heart transplantation and cardiac death have been described. There were two cases of young women, both 25 years old, classified as DMD carriers after the diagnosis of DMD in her son [30] and another due to the diagnosis of male brothers with DMD [31]. In the first case, the patient presented palpitations (due to sustained ventricular tachycardia), syncope, and dyspnea and in the second case, there was severe dyspnea during the 36th week of pregnancy. Both successfully underwent heart transplantation. It was postulated that the hemodynamic stress of pregnancy might have contributed to cardiac decompensation [31]. Another case recently described was that of a 60-year-old woman (whose son died from DMD) with a history of muscle weakness, which progressed to heart failure 6 years earlier, presenting with cardiogenic shock when she underwent a successful heart transplant [32]. However, mortality in these patients with DMD dystrophinopathy may occur due to heart failure or sudden cardiac death, with rates of 2.6% [19] and 4% [26].

## 3 CARDIAC ASSESSMENT

Due to the possibility of cardiac involvement in DMD carriers, it is recommended that they undergo clinical cardiac evaluation and through tests, such as electrocardiogram and non-invasive imaging tests, every 3 to 5 years, if asymptomatic or at shorter intervals depending on the symptoms [8, 12, 19, 33]. This screening should be carried out starting in the second or third decade of life or at the time of their children's diagnosis [8, 33]. Despite this recommendation, 25% of DMD carriers were unaware of their carrier status and, 21% of women at risk of heart disease were not subjected to any evaluation, and 37% of them did not undergo an echocardiogram in a study with 182 participants [34]. In other studies, cardiac assessment rates in DMD carriers were also low, 28.3% [35] and 35% [36].

As previously mentioned in this article, the main mechanism of clinical involvement is distorted inactivation of the X chromosome [8, 16, 17]. However, in addition to these chromosomal aberrations, simple inheritance conditions (compound heterozygotes – two different mutations acting together to produce a phenotype similar to homozygous mutant individuals) and hormonal events (complete androgen insensitivity in combination with a DMD gene mutation) can also be responsible for the manifestation of symptoms [37]. With these pathological changes, there is a higher expression of the X chromosome with the abnormal dystrophin allele. Thus, with the dysfunction of dystrophin, which stabilizes the plasma membrane, there is a loss of membrane integrity, with increased calcium influx into the cell and eventual cell death [8]. Furthermore, dystrophin deficiency leads to the displacement of neuronal nitric oxide synthase from the sarcolemma to the cytosol, with reduced nitric oxide production, resulting in functional ischemia [8, 38, 39]. Although the relationship between molecular mechanisms and the dystrophic phenotype has not been fully explained, reduced muscle performance and histological ischemic damage have been observed in experiments, requiring clinical studies [39].

One of the tests for evaluation is the electrocardiogram (ECG), however, this alone does not allow for adequate screening. Most studies also included carriers of Becker muscular dystrophy. The number of DMD carriers ranged from 16 [21] to 129 [27] women. Changes, such as T wave inversion or nonspecific changes in ventricular repolarization [20, 40], R/S ratio > 3 mm in V1 [41] have been described, as well as right bundle branch block or left bundle branch block [20]. At least one change in the ECG was observed in 41% [27] and 72% of cases [20] and the main changes were the R wave > 4 mm in V2 in 60% of cases and nonspecific changes in ventricular repolarization in 62% of cases [20].

The echocardiogram is one of the imaging tests used to assess DMD carriers. Left ventricular dilation was observed in 2.5% to 19% of female DMD carriers [18, 27, 40], but there are studies with 53 and 16 women that demonstrated systolic dysfunction in 62% and 75% of cases, respectively [20, 21]. There was no association between systolic dysfunction and age over 50 years [20], the presence of muscular symptoms, CK levels, or type of mutation [27, 41]. Furthermore, systolic dysfunction may be present in asymptomatic women. A recent study with 44 DMD carriers with a mean age of 38.8 years compared to 17 women as a control group, matched by age, demonstrated a larger left ventricular systolic diameter and segmental wall motion abnormalities in the first group, which was asymptomatic [42]. However, among 28 women with DMD dystrophinopathy, that is, with muscular or cardiac symptoms, whose ECG demonstrated an increase in the R wave in V1, 89.3% of them had an ejection fraction below 50% on the echocardiogram [43]. In relation to women with Becker muscular dystrophy, DMD carriers had a higher proportion of left ventricular involvement [41], and among those who developed dilated cardiomyopathy during the 9-year follow-up, 91% were DMD carriers, with a median age of 53 years [44].

Another non-invasive imaging test that is superior to an echocardiogram is cardiac magnetic resonance (CMR). This examination allows the assessment of volumes, left ventricular function, abnormalities of parietal contractility, and characteristics of the myocardial tissue. It can be performed with late gadolinium enhancement to quantify myocardial fibrosis [45]. Using CMR, left ventricular systolic dysfunction was detected in between 14% and 40% of DMD carriers in studies with a sample of 5 to 36 women. Myocardial fibrosis by late gadolinium enhancement was detected in 35% to 65% of DMD carriers [18]. The involvement was mainly in the inferolateral region of the left ventricle in the subepicardial portion [28]. These findings were confirmed in a larger study, with 36 female carriers of DMD/Becker muscular dystrophy (20 DMD carriers) [46]. The lateral free wall of the left ventricle was involved in 88% of cases, followed by the inferior wall in 19% of cases. The most affected segment was the inferolateral basal wall, with a subepicardial pattern in 88% of carriers. A comparison was made with 24 first-degree male relatives of these carriers, observing the same pattern of myocardial fibrosis. Thus, when cardiomyopathy was present in male patients with DMD, cardiomyopathy was also found in female carriers.

There was an association between the findings of late gadolinium enhancement and lower left ventricular ejection fraction in CMR [20, 47], in addition to the association with higher CK and CK-MB levels and shorter distance covered in the 6-min walk test [47]. Late enhancement, detected mainly in the inferolateral and lateral segments, was present in 85.7% of women with the DMD mutation and elevated pro-brain natriuretic peptide in a population of 20 carriers [47].

This predilection for involvement of the inferolateral and inferior segments, evidenced through myocardial native T1 mapping, was also demonstrated in a recent study. In this study, 38 asymptomatic female carriers of Duchenne muscular dystrophy gene mutations and 22 healthy women were included, with no difference in age or left ventricular ejection fraction between the 2 groups [48]. The explanation for both the changes on the ECG (increase in the amplitude of the R wave in V1 and/or V2), and for the more pronounced involvement of the inferolateral and inferior segments may be the same as that which has been attributed to male patients with DMD. These changes may occur due to dystrophin deficiency with impairment of Purkinje cells, resulting in inflammation, necrosis and fibrosis of cardiomyocytes in the basal wall of those segments [49].

This cardiac fibrosis can progress over time and occur at older ages; however, it is without association with skeletal muscle involvement. In a prospective cohort study, 77 DMD and Becker muscular dystrophy carriers, 22 non-carrier mothers, and 25 controls, with no difference in age, underwent cardiopulmonary exercise testing and CMR. In multivariate analysis, the predictors of late enhancement present in 48% of carriers were age (odds ratio of 10.9 per decade of age), presence of ventricular ectopy in the recovery of exercise and high CK. Despite the high serum CK levels, the maximum oxygen consumption during the exercise testing was similar between the groups [50]. This was the first study with this exercise testing evaluation, demonstrating the discordance between cardiac and skeletal muscle impairment in women carrying dystrophin mutations.

The detection of subclinical myocardial involvement can also be performed by myocardial strain analysis by CMR 3D feature-tracking. A study with 111 patients with neuromuscular diseases, including 6 DMD carriers, demonstrated a reduction in myocardial deformation rates before the development of myocardial fibrosis or ventricular remodeling [51].

The progression of myocardial fibrosis with age was demonstrated through biopsies in a longitudinal study of a 29-year-old woman at the time of her son's genetic diagnosis of DMD [52]. There was an increase in the degree of myocardial fibrosis, but the proportion of dystrophin-negative cardiomyocytes remained almost constant in biopsies performed later at 35 and 44 years of age. The authors concluded that both dystrophin-negative and dystrophin-positive cardiomyocytes can be damaged during the progression of heart failure.

Regarding other cardiac exams, such as Holter and myocardial perfusion test in DMD carriers, there are only 2 studies. In the recent study by Solheim *et al.* with 53 women with pathogenic variants of DMD, 7.5% had frequent ventricular extrasystoles (>30/hour) and 32% had ventricular bi- or trigeminy, mainly those over 50 years of age [20]. As for single-photon emission computed tomography of the myocardium with thallium-201, the examination was carried out in only 2 women with dystrophinopathy, detecting reduced blood flow in the posteroinferior wall of the left ventricle [43].

Table **1** shows the main changes in complementary exams for cardiac assessment in women with DMD mutation.

Table **2** shows data from studies on diagnosis and frequency of cardiac involvement in DMD carriers [19, 21, 27, 28, 40, 42-44, 46-48, 50, 53-56]. No studies were listed in this table in which the population also included carriers of Becker muscular dystrophy, and the data were presented together with that of female DMD carriers.

## 4 BIOMARKERS

Biological indicators can be used to support diagnosis. The serum CK test, a muscle-specific protein, is indicated to identify heterozygous women. However, its concentration may be normal in DMD carriers [9]. A recent study detected increased CK in 57% of women with pathogenic dystrophin gene variants, including DMD carriers and Becker muscular dystrophy mutation carriers [20]. Screening using the CK test can identify DMD carriers. Total CK level performed on 37,268 women of reproductive age identified 62 women with sustained serum CK >200 U/L and 16 women with a family history of DMD. The genetic test made it possible to diagnose 6 of them as definitive carriers of DMD [57]. Among carriers of female variants of dystrophinopathy, the CK level was higher in those who were symptomatic, with a value of 6,659 U/L (N=36) compared to a value of 131 U/L in those who were asymptomatic (N=104) [25]. Despite these findings, the sensitivity and specificity of serum CK levels as a biomarker in DMD carriers are 33.3% and 50%, respectively [58]. Therefore, although it can identify DMD carriers, CK screening has only modest predictive value for patients with dystrophinopathy [59]. Elevated levels of alanine transaminase and aspartate transaminase have not been considered adequate due to liver injury or underlying muscle disease [58, 59]. Thus, those with symptoms could undergo gamma-glutamyl transferase measurement to avoid unnecessary liver tests [59].

There are other cardiac biomarkers, such as cardiac troponin I and natriuretic peptides [12], whose analysis with high sensitivity tests, including longitudinal tests, has been recommended for DMD cardiomyopathy [60]. Among over 2/3 of women with a pathogenic DMD variant with cardiac involvement, the increase in this troponin occurred in 9% of women and the pro-B-type natriuretic peptide in 6% [20]. There was also an increase in pro-B-type natriuretic peptide in 66.7% of DMD carriers with late gadolinium enhancement by CMR [47]. Furthermore, levels of natriuretic peptides increased with cardiac symptoms and decreased after heart failure treatment in patients with dystrophinopathy [26], given their well-established role in diagnosis and risk stratification in heart failure. However, despite the potential of these and other biomarkers to assess disease pathogenesis and, progression and response to therapies, there is a heterogeneity of DMD [58] and additional analyses are ongoing to verify the role of some of these biomarkers [60].

Another class of biomarkers are microRNAs, which play an essential role in controlling the development of cardiac and skeletal muscle, and can allow early diagnosis of DMD, monitoring of its progression, and the effectiveness of treatment [58]. This has also been demonstrated in female DMD carriers, whose negative regulation was related to the presence of functional and/or structural cardiac abnormalities [60-62].

Table **3** shows the biomarkers and their importance in women with DMD mutation.

## 5 TREATMENT

**Fig. (1):**
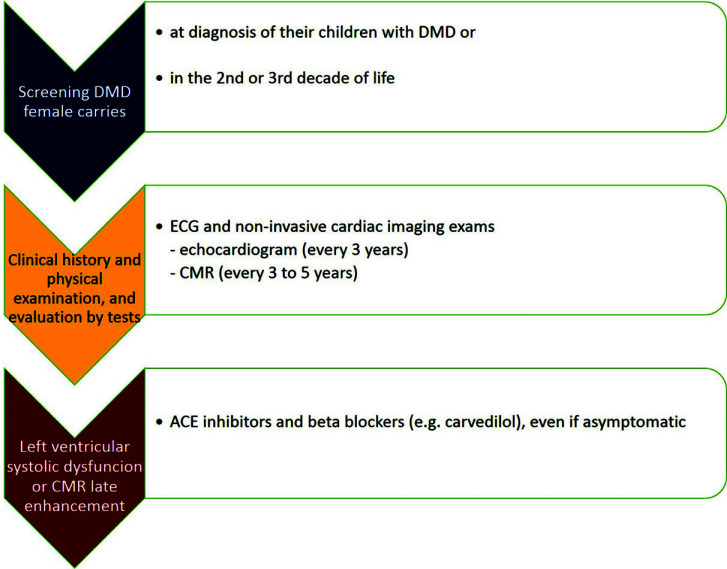
Algorithmic approach for cardiac evaluation of DMD female carriers. **Abbreviations:** DMD: Duchenne muscular dystrophy; CMR: cardiac magnetic resonance; ACE: angiotensin-converting enzyme.

The treatment of DMD carriers or women with dystrophinopathy depends on the clinical approach and ECG and imaging tests. Therefore, it is recommended that an evaluation be carried out every 3 to 5 years to detect symptoms or signs [8, 12, 19, 33], taking into account that there is a risk of developing heart disease *de novo* over time [12] (Fig. **1**). Treatment depends on the clinical stage. Although there are no large-scale clinical trials, the use of angiotensin-converting enzyme (ACE) inhibitors and beta blockers is indicated in the presence of left ventricular systolic dysfunction on echocardiography, even in asymptomatic carriers [2, 9, 18]. Angiotensin receptor blocker is indicated in case of intolerance to ACE inhibitors [9, 12]. For women undergoing CMR and with late enhancement, treatment with ACE inhibitors and mineralocorticoid antagonists is also indicated [12]. In a study with 53 DMD carriers, of which 63% had reduced longitudinal function in global longitudinal strain and 21% had an ejection fraction lower than 54%, ACE inhibitors were used by 11%, beta-blockers by 4%, and diuretics by 13% of the women. In this study, only 15% reported dyspnea, a proportion with no difference in relation to the 20 non-participants interviewed [20]. For women with refractory heart failure, heart transplantation, as previously discussed in this article, can be performed successfully [30-32].

Thus, the guidelines already established for the approach and treatment of patients with heart failure [63, 64], according to their stage and left ventricular ejection fraction, must also be followed for these women with DMD mutation. There are no studies yet on the use of angiotensin receptor–neprilysin inhibitors and sodium-glucose co-transporter 2 inhibitors for these women. Although, there are also no studies on the use of ivabradine. There is a case report of the use of this medication in a woman with DMD dystrophinopathy with exertional dyspnea and systolic dysfunction who could not tolerate beta-blockers [65]. Furthermore, the multidisciplinary approach, including a geneticist, neurologist, and physiotherapist, as well as psychosocial support, is of paramount importance.

## 6 FUTURE DIRECTIONS

For better knowledge about cardiac involvement in DMD carriers, a more comprehensive registry of these women is necessary. There is The Duchenne Registry in which only 18% are enrolled as DMD carriers (https://www.duchenneregistry.org/). These registries may bring understanding to the variation in phenotypic expression, as well as the possibility of other presentations such as Takotsubo cardiomyopathy. It is recommended that other members of the affected family, such as daughters, sisters, and others at risk and undergoing genetic testing, be included in this registry.

Emerging techniques for a more refined assessment, such as myocardial strain from 3D imaging [66], may allow for early identification and stratification of cardiac involvement, with repercussions on the approach to these patients.

Clinical trials with heart failure drugs and gene therapy for DMD carriers are also needed to evaluate their efficacy and safety, with a positive impact on survival.

## CONCLUSION

The prevalence of DMD carriers was 1 in 1046 women. The majority of these women are asymptomatic. However, cardiac involvement can be verified in between 2.5% and 75% of them, through echocardiography. There is a predilection for involvement of the inferolateral and inferior segments of the left ventricle. This results in an increase in the amplitude of the R wave in V1 and/or V2 due to myocardial fibrosis, also detected by CMR. This is the same pattern detected in male first-degree relatives of these carriers. Some biomarkers, such as cardiac troponin and microRNA, may be associated with myocardial fibrosis. Clinical assessment and thorough tests are recommended every 3 to 5 years to detect symptoms or signs. The treatment of women with the DMD mutation follows the same guidelines for patients with heart failure.

## AUTHORS’ CONTRIBUTIONS

The author confirms sole responsibility for the following: study conception and design, data collection, analysis and interpretation of results, and manuscript preparation.

## Figures and Tables

**Table 1:** Main changes in ECG, echocardiogram and CMR in women with DMD mutation.

**Complementary Exam**	**Main Changes**
ECG	↑ R/S ratio V1, V2
Echocardiogram	Left ventricular systolic dysfunction in up to 75% of cases
CMR	Myocardial fibrosis in up to 65% of cases, especially in the inferolateral segment and subepicardial pattern

**Abbreviations:** ECG: electrocardiogram; CMR: cardiac magnetic resonance.

**Table 2:** Cardiac evaluation studies in DMD carriers, with data on sample size, age, diagnostic methods, and frequency of cardiac involvement.

**First Author, Year [Reference]**	**Number of DMD Carriers**	**Age DMD Carriers (Years)**	**Methods**	**Cardiac Involvement**
Ueda Y, 1995 [21]	16	-	ECG, echo (in 16), Tl-201 myocardial SPECT scan (in 2 carriers)	↓EF in 75% of carriers, hypoperfusion in the inferior-posterior wall
Politano L, 1996 [19]	152	5 to 60	ECG, echo, myocardial scintigram	Dilated cardiomyopathy in 8.6%
Hoogerwaard EM, 1999 [27]	85	18 to 58	ECG, echo	Dilated cardiomyopathy in 8.2%, ↑LV in 18.8%
Grain L, 2001 [40]	56 (35 controls)	45.0 (median)	ECG, echo	Abnormal echo in 9 carriers
Ywase T, 2010 [28]	7	54.0 (mean)	ECG, echo, CMR	EF 28% (in 1), late enhancement in inferolateral segments (in 4)
Schade van Westrum SM, 2011 [44]	60	32 to 68	ECG, echo	Dilated cardiomyopathy in 3%
Mavrogeni S, 2013 [53]	25	48.0	ECG, echo, CMR	LV dysfunction in 24%, late enhancement in 18 carriers
Giglio V, 2014 [54]	30 (37 controls)	36.0	ECG, echo, CMR	Late enhancement in 46.6%
Schelhorn J, 2015 [55]	15	32.3	CMR	↓EF in 33%, late enhancement in 60%
Lang SM, 2015 [56]	22	40.9	CMR	LV dysfunction in 18%, late enhancement in 35%
Florian A, 2016 [46]	20	40.0 (mean)	CMR	Late enhancement in 65%
Wexberg P, 2016 [47]	20	39.4 (mean)	ECG, echo, CMR	Late enhancement in 45%
Adachi K, 2018 [43]	28	47.8 (mean)	ECG, echo (in 25), CMR (in 6)	EF < 50% in 32.0% of carriers, low strain in the basal to mid-posterior wall
Kincl V, 2020 [42]	44 (17 controles)	38.8 (mean)	echo and tissue Doppler imaging	↑ LV end-systolic dimension
Mah ML, 2020 [50]	77 (22 controls)	41.3 (mean)	Cardiopulmonary exercise testing, CMR	EF 59.2% (mean), late enhancement in 49%
Masárová L, 2023 [48]	38 (22 controls)	39.1	CMR	Higher mean extracellular volume values in the inferior and inferolateral segments

**Abbreviations:** ECG: electrocardiogram; Echo: echocardiography; EF: ejection fraction; LV: left ventricle.

**Table 3:** Biomarkers and their importance in DMD carriers.

**Biomarkers**	**Importance**
CK	Modest predictive value
Cardiac troponin I and natriuretic peptides	High Sensitivity Tests in DMD cardiomyopathy
MicroRNAs	Reduced levels correlate with the presence of myocardial fibrosis on CMR

**Abbreviations:** CK: creatine kinase; CMR: cardiac magnetic resonance.

## LIST OF ABBREVIATIONS

ACE: Angiotensin-Converting Enzyme
CK: Creatine Kinase
CMR: Cardiac Magnetic Resonance
DMD: Duchenne Muscular Dystrophy
ECG: Electrocardiogram
